# Tuning microbial hosts for membrane protein production

**DOI:** 10.1186/1475-2859-8-69

**Published:** 2009-12-29

**Authors:** Maria Freigassner, Harald Pichler, Anton Glieder

**Affiliations:** 1 Institute of Molecular Biotechnology, Graz University of Technology, Petersgasse 14, 8010 Graz, Austria; 2 Austrian Centre for Industrial Biotechnology (ACIB), c/o Research Centre Applied Biocatalysis, Petersgasse 14, A-8010 Graz, Austria

## Abstract

The last four years have brought exciting progress in membrane protein research. Finally those many efforts that have been put into expression of eukaryotic membrane proteins are coming to fruition and enable to solve an ever-growing number of high resolution structures. In the past, many skilful optimization steps were required to achieve sufficient expression of functional membrane proteins. Optimization was performed individually for every membrane protein, but provided insight about commonly encountered bottlenecks and, more importantly, general guidelines how to alleviate cellular limitations during microbial membrane protein expression. Lately, system-wide analyses are emerging as powerful means to decipher cellular bottlenecks during heterologous protein production and their use in microbial membrane protein expression has grown in popularity during the past months.

This review covers the most prominent solutions and pitfalls in expression of eukaryotic membrane proteins using microbial hosts (prokaryotes, yeasts), highlights skilful applications of our basic understanding to improve membrane protein production. Omics technologies provide new concepts to engineer microbial hosts for membrane protein production.

## Background

We have seen amazing advances in the field of membrane protein research over the past four years. For the first time, high resolution structures for pharmaceutically relevant eukaryotic membrane proteins, including class A G-protein coupled receptors (GPCR) [1-5], transporters [6] and channel proteins [7-16] became available and provided valuable insight into their mode of action (Table 1). A few years earlier, structures of the soluble domains of human CYP3A4 and CYP2D6 of the Cytochrome P450 family, the most important drug metabolising enzymes, had been solved [17-20]. Actually, many years of strenuous efforts were required to get the proteins to crystals, relying on iterative optimization of all steps from expression to purification and crystallization. The total number of membrane protein structures deposited in the protein data bank (PDB) also strikingly reflects that membrane protein research can not yet keep pace with the one of soluble proteins. Among over 58,000 total entries, only some 100 of all coordinate sets belong to membrane proteins [21]. Nature, however, provides plenty of different targets: roughly one third of all open reading frames encode for membrane proteins, as predicted for fully sequenced genomes of eubacterial, archaean and eukaryotic organisms [22,23]. The discrepancy between biodiversity and poor structural knowledge can be largely attributed to the low natural expression of membrane proteins, to their hydrophobic character which complicates overexpression of functional membrane proteins, as well as to difficulties during their purification and crystallization.

**Table 1:** Eukaryotic membrane proteins with high resolution structures.

Protein	Expression Host	PDB coordinates	Resolution (Å)	Reference
**TRANSMEMBRANE PROTEINS: BETA-BARREL**				
**Beta-Barrel Membrane Proteins: Mitochondrial Outer Membrane**				
Human VDAC-1 voltage dependent anion channel	*E. coli*	2K4T^1^, 2JK4^2^	4	[210,211]

Murine VDAC-1 voltage dependent anion channel	*E. coli*	3EMN	2.3	[212]

**TRANSMEMBRANE PROTEINS: ALPHA-HELICAL**				

**G Protein-Coupled Receptors**				

Rhodopsin: Bovine Rod Outer Segment mutant N2C/D282C	COS cells	2J4Y	3.4	[122]

Engineered turkey β_1 _adrenergic receptor	*Trichoplusia ni*	2VT4	2.7	[1]

Human β_2 _adrenergic receptor, from β_2_AR365-Fab5 (2R4R) and β_2_AR24/365- Fab5 complexes (2R4S)	*Spodoptera frugiperda*	2R4R, 2R4S	3.4/3.7	[2]

Engineered human β_2 _adrenergic receptor	*Spodoptera frugiperda*	2RH1, 3D4S	2.4/2.8	[3,4]

Human A_2A _adenosine receptor, In complex with a high-affinity subtype-selective antagonist ZM241385.	*Spodoptera frugiperda*	3EML	2.6	[5]

**Integrin Adhesion Receptors**				

Human Integrin αIIbβ3 transmembrane-cytoplasmic heterodimer, NMR Structure	*E. coli*	2KNC		[213]

**Snare Protein Family**				

Syntaxin 1A/SNAP-25/Synaptobrevin-2 Complex from *Rattus rattus*	*E. coli*	3HD7	3.4	[214]

**Ion Channels**				

Kir3.1-Prokaryotic Kir Chimera: *Mus musculus & Burkholderia xenovorans*	*E. coli*	2QKS	2.2	[7]

ASIC1 Acid-Sensing Ion Channel: *Gallus gallus*; 2QTS: N- and C-terminal deletions, 3HGC: minimal functional channel	*Spodoptera frugiperda*	2QTS, 3HGC	1.9/3.0	[8,215]

ATP-gated P2X4 ion channel (apo protein): Danio rerio (zebra fish) (expressed in SF9 cells), 3.1 Å; Closed state. A construct, 3.5 Å: 3I5D	*Spodoptera frugiperda*	3H9V, 3I5D	3.1/3.5	[216]

Kv1.2 Voltage-gated potassium Channel: *Rattus norvegicus*	*Pichia pastoris*	2A79	2.9	[9]

Kv1.2/Kv2.1 Voltage-gated potassium channel chimera: *Rattus norvegicus*	*Pichia pastoris*	2R9R	2.4	[10]

**Other Channels: Aquaporins and Glyceroporins**				

Rat AQP4 aquaporin water channel, S180D mutant (2ZZ9)	*Spodoptera frugiperda*	2D57, 2ZZ9	3.2/2.8	[11,12]

Human AQP4 aquaporin water channel	*Pichia pastoris*	3GD8	1.8	[13]

Human AQP5 aquaporin water channel	*Pichia pastoris*	3D9S	2.0	[14]

Plant SoPIP2;1 aquaporin: *Spinacia oleracea *in closed (1Z98) and open conformation (2B5F)	*Pichia pastoris*	1Z98, 2B5F	2.1/3.9	[15]

*Pichia pastoris *AQY1 aquaporin, pH 3.5 (2W2E) and 8.0 (2W1P)	*Pichia pastoris*	2W2E, 2W1P	1.15/1.40	[16]

**Other Channels: Gap Junctions**				

Human Connexin 26 (Cx26; GJB2) gap junction	*Spodoptera frugiperda*	2ZW3	3.5	[217]

**Membrane-Associated Proteins in Eicosanoid and Glutathione Metabolism**				

Human Microsomal Prostaglandin E Synthase 1: Human (Electron Diffraction) In complex with glutathione.	*E. coli*	3DWW	3.5	[218]

*Human *5-Lipoxygenase-Activating Protein (FLAP) with Bound MK-591 Inhibitor (2Q7M), FLAP with iodinated MK-591 analog (2Q7R)	*E. coli*	2Q7M, 2Q7R	4.0	[219]

*Human *Leukotriene LTC_4 _Synthase in complex with glutathione	*S. pombe*	2PNO	3.3	[220]

*Human *Leukotriene LTC_4 _Synthase in complex with glutathione/apo form	*Pichia pastoris*	2UUH, 2UUI	2.15/2.0	[67]

**ATP Binding Cassette Transporters**				

*Mus musculus *P-Glycoprotein (3G5U), With bound QZ59-RRR (3G60) and QZ59-SSS (3G61)	*Pichia pastoris*	3G5U, 3G60, 3G61	3.8/4.4/4.35	[6]

**P-type ATPase**				

Human Na,K-ATPase Regulatory Protein FXYD1	*E. coli*	2JO1^1^		[221]

Phospholamban homopentamer: Human sarcoplasmic reticulum	*E. coli*	1ZLL^1^, 1FJK^1^, 1FJP^1^		[222]

Plasma Membrane H^+^-ATPase: *Arabidopsis thaliana*	*S. cerevisiae*	3B8C	3.6	[223]

^1 ^Structure determined by NMR spectroscopy,

^2 ^Structure determined by combining X-ray and NMR data.

Paradoxically, from a pharmaceutical point of view, membrane proteins - in particular GPCRs and transport proteins - constitute key drug targets, as most signal transduction processes are initiated or transmitted via membrane proteins. The facts that at least 50% of all commercially available drugs target membrane proteins, that the major enzymes initiating drug metabolism are microsomal cytochrome P450 enzymes and that the human immune defense against invading pathogens is often initiated by their membrane proteins [24,25], underline the importance of elucidating three-dimensional structures of membrane proteins [26]. Evidently, great efforts are made to better understand membrane proteins from a structural and functional point of view. Unfortunately, (recombinant) expression of eukaryotic membrane proteins often suffered from low expression levels, instability of proteins and/or degradation by the host's proteolytic machinery. While in the past many labs have focused on their single targets, there was a strong trend towards studying a large number of different proteins simultaneously more recently. In this regard, many consortia and research groups have tried to rationalize all processes from expression to purification and crystallization, hoping to end up with at least a few membrane proteins that are amenable to crystallization (structural genomics). Whatever route is taken, it is obvious that improvements in early steps, particularly concerning expression levels of functional membrane proteins are of tremendous benefit to subsequent experiments, especially protein purification, characterization and crystallization.

## Review

### 1. Expression systems

In the past, unreasonably more structures have been solved for bacterial and archaebacterial membrane proteins than for eukaryotic ones (Database of Membrane Proteins of Known Structure [21]). Early success in crystallization of eukaryotic membrane proteins came from the use of native proteins [27-31]. However, this was only successful if a uniquely rich source was available, e.g. the retina providing mg amounts of rhodopsin, and it precluded protein modifications, e.g. isotope labelling for NMR analysis. Due to difficulties in isolating native membrane proteins in sufficient quantity and quality, strong efforts were made towards establishing heterologous expression systems for the production of eukaryotic membrane proteins. Prior to 2005, no high resolution structure had been solved for any recombinant eukaryotic membrane protein. Since then, 28 unique structures have been elucidated for eukaryotic membrane proteins (Table 1). Among microbes, the prokaryote *E. coli *and the yeast *Pichia pastoris *have been most successfully used in production of eukaryotic membrane proteins for structural analyses.

#### 1.1. Prokaryotic expression systems and their limitations in hosting eukaryotic membrane proteins

*E. coli *still tops the list of the most popular expression systems for prokaryotic and eukaryotic membrane proteins, given the broad choice of molecular biology tools and strains available [32]. Its ease of handling, especially in a high-throughput way, enabling analyses of many proteins in parallel, sophisticated genetic manipulation techniques, the availability of membrane protein-adapted strains, but also cost effectiveness - all these factors contribute to the popularity of *E. coli *and its preferential use in structural genomics initiatives targeting membrane proteins of both prokaryotic and eukaryotic origin. While *E. coli *provides an optimal environment for prokaryotic proteins, its capability to host eukaryotic membrane proteins was limited, however [26,33]. Although reasonable success in providing eukaryotic membrane proteins for structural analyses has been documented (Table 1), success rates are low compared to eukaryotic expression hosts. Often, prokaryotic homologues are studied in lieu of eukaryotic membrane proteins. However, only 13% of all membrane protein families are common between prokaryotes and eukaryotes [34].

For eukaryotic proteins, prokaryotic folding pathways might be far from optimal, as subtle differences between assembly machineries impede their correct processing. While the eukaryotic translocon possesses charged residues mediating interaction with the nascent polypeptide chain during translocation and insertion into the membrane, bacterial counterparts are lacking these features [35]. Additionally, the membrane composition of different eukaryotic organellar membranes is different from the prokaryotic cytoplasmic membrane and also processing of premature proteins often fails in prokaryotes, likely leading to mistargeting or misfolding of eukaryotic membrane proteins [36]. Apart from incompatibilities in protein processing, varying polypeptide elongation and folding rates contribute to the obstacles experienced in heterologous expression of eukaryotic membrane proteins in prokaryotic hosts [37]. The rate of polypeptide chain elongation is 4 to 10 times faster in prokaryotes compared to eukaryotic cells. As all steps during translation, folding and membrane insertion need to be properly balanced allowing the protein to find its native conformation, high translation rates might lead to exposure of hydrophobic segments, and thus promote their aggregation. In the past, membrane protein expression was performed just like expression of simple soluble proteins. Strong promoter systems have been preferentially employed to drive expression, e.g. T7 promoter, thereby obviously exceeding the translocation machinery's ability to correctly process the enzyme [38]. As a consequence of high transcript abundance combined with high prokaryotic translational rates, eukaryotic membrane proteins often accumulate in inclusion bodies [26,39]. At first view, this offers some apparent advantages such as no or reduced toxicity caused by heterologously expressed proteins, protection against proteases and, thus, high yields. However, inclusion bodies require renaturation, which is anything but trivial. Although some membrane proteins have been successfully refolded from inclusion bodies [40], *in vitro *renaturation remains a contentious issue, as many proteins fail in reaching the native, functional conformation once they are denatured. Refolding has been most successfully employed for β-barrel type membrane proteins, which are innately more robust than α-helical membrane proteins, but also not as sought after as the latter [41]. Therefore, expression of properly folded and membrane-embedded proteins is highly desirable, be it by attenuating expression strength, e.g. by the use of weak promoters and low copy number, be it by modifying cultivation conditions, e.g. growth temperature and inducer concentration, as will be discussed further below [38,42]. In the case of low temperature, the effect of reduced transcription and translation rates seems to combine with the availability of additional chaperones and foldases [43].

Overexpression of eukaryotic proteins is furthermore often complicated due to *E. coli*'s inability to perform posttranslational modifications that can be crucial for proper folding, targeting and function [44]. Many eukaryotic membrane proteins rely on the presence of specific post-translational modifications (PTM) to attain full activity, e.g. acylation and phosphorylation, or to retain stability mainly by glycosylation [33]. *E. coli *is not able to glycosylate proteins and, thus, not the host of choice if such modification is crucial for activity [33,44,45]. On the other hand, non-glycosylated proteins are usually rather homogeneous and uniform, which might be advantageous for experiments depending on very pure enzymes, e.g. crystallization. Often, phosphorylation events are necessary for protein activation e.g. triggering conformation changes as observed for many GPCRs, thus precluding *in vivo *studies [46,47]. Further, *E. coli *lacks endogenous G proteins impeding its use for functional studies of GPCRs, but coexpression [48] or even external addition of the G protein α subunit to the membrane preparation was able to restore high-affinity binding properties [49].

According to the current view, membrane proteins exist as protein-lipid complexes with specific lipids inevitably required to promote folding, retain activity and confer structural stability, as can be seen from their presence in many crystal structures [36,50,51]. Bacterial membranes are devoid of sterols and derivatives, polyunsaturated fatty acid chains and sphingolipids, which are known to play a key role in folding of membrane proteins in eukaryotic membranes (reviewed in [36]). Failure to produce functional mammalian receptors in *E. coli *has thus been partly ascribed to the lack of essential lipids, e.g. cholesterol during expression of mammalian serotonin transporter [52,53]. Many mitochondrial membrane proteins tightly interact with cardiolipin, and large amounts of F_1_F_0 _ATP synthase were only obtained after modulating lipid biosynthesis pathways and, thus, lipid composition of *E. coli *membranes towards an increased cardiolipin content [54]. In a few cases, the addition of lipids during expression and purification restored protein activity [36].

Although reasonable expression levels have been demonstrated for different individual eukaryotic membrane proteins [42], the most successful attempts have been found for β-barrel type membrane proteins (Table 1). Expression levels were typically several orders of magnitude lower compared to their bacterial homologs [52].

Besides *E. coli*, the gram-positive bacterium *Lactococcus lactis *has emerged as alternative prokaryotic host due to a relatively small number of endogenous membrane proteins and thus high capacity to host foreign proteins [55-58]. As facultative anaerobic bacterium, *L. lactis *is able to reach high cell densities even under anaerobic conditions. Being surrounded by a single membrane, *L. lactis *cells can be broken easily, thereby facilitating downstream processes. Due to the relatively small genome i.e. approx. 50% of *E. coli*, however, some auxiliary proteins and chaperones with essential functions in folding and membrane insertion might not be provided by *L. lactis *[57]. While 21 of 25 (84%) prokaryotic transport proteins were successfully expressed in *E. coli*, only 10 thereof (40%) could be obtained in *L. lactis *cells, as demonstrated recently [59]. On the other hand, the proportion of functional protein of prokaryotic origin was higher in *L. lactis *compared to *E. coli *[60]. Though levels of eukaryotic membrane proteins lag still behind those obtained for prokaryotic ones, yields are comparable to those obtained in *E. coli *[57]. In light of the fact that formation of inclusion bodies has not been observed so far during expression of membrane proteins, *L. lactis *provides an attractive alternative to *E. coli *[55,57].

The halophilic archaeon *Halobacterium salinarum *also ranges among promising non-standard expression hosts due to its elaborate membrane system and, thus, its high capacity to host additional membrane proteins. Nonetheless, high level expression seemed to be limited to bacteriorhodopsin and derivatives [61,62]. Among prokaryotic hosts, the photosynthetic bacteria *Rhodobacter sphaeroides *and *Rhodobacter capsulatus *provide plenty of membrane space while concomitantly offering elaborate and highly efficient insertion machineries. During prototrophic growth, they use light for energy production and growth, and thereby enrich intracytoplasmic membranes [63-65]. However future experiments will have to demonstrate whether this is sufficient to obtain high yields of membrane proteins.

#### 1.2. Yeasts as expression systems for eukaryotic membrane proteins

Despite individual breakthroughs, prokaryotic expression systems tend to provide an inhospitable environment for eukaryotic membrane proteins. Therefore, most recent attempts were directed towards employing eukaryotic hosts for eukaryotic membrane protein expression. In this context, yeasts are an inexpensive, yet efficient alternative to prokaryotes. Providing a eukaryotic environment, yeast cells are capable of processing proteins similarly to higher eukaryotes. Simple handling, rapid growth and powerful genetic tools contributed to their popularity [66]. To date, 10 out of 28 unique structures of heterologously expressed eukaryotic membrane proteins were obtained from yeast-derived material, including the best-resolved structures (1.8 - 2.0 Å) of mammalian membrane proteins (Table 1) [13,14,67].

Most commonly, membrane proteins are overexpressed and targeted to the ER membrane from where they are transported to the plasma membrane in vesicles [68]. Unlike *E. coli*, yeasts are able to perform various post-translational modifications including proteolytic processing of signal sequences of both pre- and prepro-type, disulfide-bond formation, acylation, prenylation, phosphorylation and certain types of O- and N-linked glycosylation that all might be essential for activity and correct folding (reviewed in [66]). Methylotrophic yeasts are an attractive alternative to *S. cerevisiae *as overexpressed proteins are less frequently hyper-mannosylated than in *S. cerevisiae*. In addition, the presence of a terminal α-1,2-mannose residue abolishes their allergenic potential [69,70]. Meanwhile, even strains with a more uniform and also with a human glycosylation pattern are available for functional studies [71-74]. Although the lipid composition of yeast membranes is closer to higher eukaryotes than to *E. coli*, the lack of certain lipids, in particular sterols, e.g. cholesterol for mammalian proteins, sito-, stigma- and campesterol for plant enzymes, might affect protein functionality. Ergosterol, the predominant endogenous sterol in fungi, might compensate for specific sterols, as shown for mammalian GPCRs and transport proteins, but for full activity presence of cholesterol might be essential [33,75]. During purification, protein activity and stability can be retained or even restored by addition of defined lipids. In this respect, cholesterol hemisuccinate was crucial in stabilizing the A_2_a receptor during purification following overexpression in *S. cerevisiae *[76].

Being the model eukaryote, ***S. cerevisiae ***has traditionally been most widely employed for the expression of eukaryotic membrane proteins [77-79]. This popularity can be largely ascribed to elaborate, yet easy genetic manipulation techniques, as documented by the availability of numerous expression plasmids. Furthermore, the vast number of available strains including entire deletion libraries allows to carry out functional complementation *in vivo*, as exemplified recently for different plant transporter proteins [80-82]. Most frequently, inducible promoters (e.g. GAL1 or GAL10) are employed to tune membrane protein expression by yeast as for 93% of membrane proteins constitutive expression resulted in impaired growth during their homologous overexpression [83,84]. In contrast to other yeasts, protocols have been established for *S. cerevisiae *to enable its use in high-throughput (HTP)-expression studies of eukaryotic membrane proteins [77-79]. While on the one hand a C-terminal GFP-His_8_-fusion is employed to assess expression levels and optimize purification [77,78], other methods rely on the combination of C-terminal His_10_- and N-terminal FLAG-tags [79], using immunodetection for estimation of expression levels. Most commonly, episomal plasmids were used for expression. Ability of *S. cerevisiae *to perform *in vivo *recombination allows ligation of the respective gene into the expression plasmid *in vivo *[77-79]. Additionally, *in vivo *cloning permits the simple construction of protein variants, as shown recently for mouse-TRPM5 channel [85], and, therefore, directed evolution of membrane proteins or selected domains.

Due to its tendency to hypermannosylate proteins and complex handling of *S. cerevisiae *in fermenter cultures, much attention has recently focused on non-conventional yeasts including *Schizosaccharomyces pombe*, *Yarrowia lipolytica, Hansenula polymorpha *and *Pichia pastoris*.

The fission yeast ***Schizosaccharomyces pombe ***outperforms other yeast species in expressing mammalian proteins with mammalian-like core glycosylation pattern and by its ability to process intron-containing genes [86,87]. Thus, *S. pombe *is a promising, yet easy-to-handle alternative to conventional yeast species with a high potential for the expression of complex proteins, e.g GPCRs [88]. *S. pombe*'s suitability to express functional eukaryotic membrane proteins was supported by a previous study on human D_2S _dopamine receptor, resulting in a five fold higher binding affinity of isolated membranes and, thus, a higher proportion of functional, membrane-integrated receptor compared to the protein produced in *S. cerevisiae *[89].

Frequently, heterologous systems are used for membrane protein expression to obtain sufficient amounts of those proteins for structural and functional studies *in vitro*. Apart from purification-oriented purposes, eukaryotic membrane proteins such as enzymes of the cytochrome P450 family are also overexpressed in heterologous systems to perform whole-cell biotransformations on highly valuable substances (reviewed in [90]). While initially bioconversions had been realized using *S. cerevisiae *[91-97], meanwhile also *S. pombe *has been successfully employed for mammalian cytochrome P450-mediated reactions [98-102]. As P450s act in concert with an electron-donating system, coupling efficiency and thus substrate conversion rates can be increased by coexpression of the respective mammalian redox partners or by protein engineering of the biocatalyst [103]. The origin and the quantity of the coexpressed reductase may influence the expression of the heme domain and the resulting activity of these complex enzymes. This is usually regulated by gene copy numbers or by the choice of the employed promoters.

Similarly, another non-conventional yeast species, ***Yarrowia lipolytica***, is emerging as host for biotransformations of hydrophobic substrates as it tolerates organic solvents and metabolizes aliphatic compounds. *Y. lipolytica *has been employed for production of cytochrome P450 enzymes, including bovine P450 17α (CYP17A) [104], human P450 CYP1A1 [105] and CYP53B1 from *Rhodotorula minuta *[106]. From these studies evidence emerged that integration of multiple expression cassettes had a positive effect on heterologous expression as observed by increased conversion rates.

Among non-*Saccharomyces *yeasts, the methylotrophic yeast ***Pichia pastoris ***has been most extensively and successfully employed in membrane protein research (Table 1). Exceptionally high cell densities can be reached during cultivation, concomitantly guaranteeing efficient and economically sustainable protein production (reviewed in [107,108]). Methylotrophic yeasts may derive all energy for growth from the utilization of methanol as carbon source by inducing expression of methanol-metabolizing enzymes, namely alcohol oxidase (AOX) and dihydroxyacetone synthase (DHAS) [109]. Because promoters of the methanol utilization pathway are strong but also tightly regulated, these are most commonly employed to drive heterologous protein expression [110,111], allowing to reach up to 90 mg L^-1 ^of membrane proteins exemplified by a human aquaporin [112]. Furthermore, protein production capacity is not compromised during growth on minimal media, which makes this yeast attractive for the production of stable isotope labelled proteins for NMR-based structural biology [113] and also for reliable industrial production. Apart from well-known advantages for protein expression, such as eukaryotic proteolytic processing, protein folding, disulfide bond formation and glycosylation, another factor contributing to *Pichia pastoris *outstanding popularity is its ability to secrete heterologous proteins while secreting very low levels of endogenous proteins. This allows extracellular accumulation of heterologously expressed soluble proteins, but also offers a route for production of eukaryotic membrane proteins as well, as they are efficiently trafficked through ER and Golgi to the plasma membrane.

Versatility of *Pichia pastoris *as host for membrane protein expression has been documented by the number of structures elucidated with *Pichia*-derived material (Table 1). In particular, a success rate of 93,5% has been found for expression of GPCRs, whereas more than half of all 100 proteins tested failed to be properly expressed in *E. coli *[26]. A closer analysis of specific binding activities also demonstrated that *Pichia pastoris *can keep up with higher eukaryotic systems, e.g. *Semliki Forest *virus-mediated expression in mammalian cells, but also showed that the ideal host is target dependent.

Thus, using complementary hosts allows to expand the expression space. If eukaryotic membrane proteins fail to be properly expressed in yeasts, attention is usually drawn to higher eukaryotic hosts, e.g. insect cells, mammalian cells or cell-free expression systems [114-116]. These expression hosts are not covered by this review, but are outlined in detail in recent articles[26,117,118].

### 2. Strategies for membrane protein overexpression and problems encountered therein

Like for soluble proteins, each membrane protein behaves in an individual, unfortunately unpredictable way upon overexpression. In previous studies, no correlation between the "expressability" of a membrane protein and protein specific parameters, i.e. size, number of transmembrane helices, hydrophobicity, was found during homologous overexpression of 300 membrane proteins in *E. coli *([119], reviewed in [120]). Other studies pointed out that heterologous expression of GPCRs in *E. coli *is limited to a size of 54 kDa and below [26]. Similarly, in *S. cerevisiae*, one of the most significant factors affecting the expression level was considered to be the molecular size of the protein - smaller proteins (< 60 kDa) generally tend to be better expressed than larger ones, as judged from homologous overexpression of more than 1000 membrane proteins in *S. cerevisiae *[84]. White and coworkers also found an inverse correlation between the number of transmembrane helices and the expression level, but presence of large extramembraneous domains might ameliorate a membrane protein's performance [84]. Highest expression levels were obtained for proteins with less than five transmembrane segments - preferentially composed of hydrophobic residues and lacking charged amino acids. Recently, in a "discovery-oriented" selection process, 234 out of 384 endogenous integral membrane proteins representing every IMP Pfam family within the yeast genome were successfully expressed under control of the GAL1 promoter in *S. cerevisiae *corresponding to a success rate of 61% with expression levels ranging from 0.5 to 5.8 mg L^-1 ^[79]. Furthermore 25% of all candidate proteins were amenable to purification using n-Dodecyl-β-D-maltoside, as judged from immunoblotting of membrane extracts and the protein's performance during size-exclusion chromatography. Like in previous studies, the success rate for expression of integral membrane proteins was tightly linked to their specific properties. Best expression levels were obtained for small proteins (MW < 100 kDa) with a low number of transmembrane helices (<6) and a low average hydrophobicity index [79]. For heterologous proteins comparative studies are missing. However, a general trend was that proteins perform better during heterologous expression when related homologous proteins are present in the respective expression host [121]. However, most strategies towards optimization of membrane protein expression focused on modifying the protein itself and optimizing cultivation conditions rather than engineering the expression host.

#### 2.1. Engineering membrane proteins for increased stability, affinity or activity

Improvements in expression levels can also be achieved by modifying the membrane protein. For the purpose of crystallization, the protein structure can be stabilized by increasing rigidity, e.g. through introduction of disulfide bonds [122] or N- and/or C-terminal truncations [11]. Furthermore, target proteins may be tailored by incorporation of point mutations with the aim to destroy biological activity and, thus, to diminish toxicity during heterologous overexpression [123]. In this regard, directed evolution strategies have turned out to be more straightforward compared to rational engineering, as the latter relies on structural information which is still scarce for membrane proteins. By employing directed evolution, expression levels, stability and binding selectivity of detergent-solubilized membrane proteins have been improved [124,125]. Variants of the turkey β_1_-adrenergic receptor with improved conformational homogeneity in presence of antagonist, enhanced tolerance to short-chain detergents and thermostability have been obtained by alanine scanning followed by in-depth mutagenesis and combination of hot spots in *E. coli *[126].

To date, screening for improved variants or enhanced expression level has been almost exclusively carried out in *E. coli*, by employing immunodetection (colony-filtration blot of membranes) [127], FACS in the presence of specific fluorescent ligands [125] or specific activity [124] and ligand binding assays [126,128]. Like for soluble proteins, activity-based screens allow to select for functional mutants [124], while screens relying on total expression levels including all immunodetection-based assays might preferentially enrich inactive, hence non-toxic proteins. Use of membranes instead of whole cells in combination with activity assays can circumvent the specified troubles, as suggested by Molina and coworkers [129]. Following initial screening using *E. coli*, selected variants are transferred to eukaryotic systems for production purposes [125]. Besides their impact on protein stability and function, mutations might directly affect transcriptional, translational and/or folding efficiency. In respect to differences in cellular milieus in between species, many promising candidates might fail in providing the same properties they have been selected for during the initial screening in prokaryotes. Recently, the suitability of *S. cerevisiae *to perform directed evolution experiments has been demonstrated, by improving ligand sensitivity of a human UDP-glucose receptor [130]. Meanwhile, the availability of protocols paves the way for the use of various yeast species as hosts in directed evolution experiments [85,131].

#### 2.2. Adaptation of the host to membrane protein production

##### 2.2.1. Relieving metabolic stress evoked by membrane protein overexpression by adjusting transcription and translation efficiency to membrane protein folding

Apart from engineering proteins of interest, expression of membrane proteins can be fine-tuned on different cellular levels in order to improve yields. During production of membrane proteins high gene dosage strategies often fail in providing more functional protein but rather entail impaired growth or accumulation of aggregated protein in inclusion bodies as a result of limited cellular capacity to correctly translocate, assemble, modify and integrate proteins into the membrane [39,132-135]. As transcript abundance does not limit membrane protein production [136], assembly of membrane proteins often works better when synthesis rates are slowed down to better match the rates of insertion and assembly. Frequently, a high copy number entails an increase in total yield, but not in amounts of functional protein, as a consequence of improper balance of protein biosynthesis and folding [135,137,138]. Low transcriptional rates can be achieved by lowering the gene dosage or switching to weak, yet tightly regulated promoters, thereby avoiding accumulation of aggregated protein [38]. As a consequence of membrane protein production, cell growth is often retarded, thus inducible promoter systems may be superior to constitutive ones in membrane protein production (e.g. P_BAD _and P_T7 _for *E. coli*, P_AOX1 _for *Pichia pastoris*), as they allow unimpaired cell growth to high densities prior to protein expression. Apart from low gene dosage or weak promoters, expression kinetics can be fine-tuned by shortening induction periods, reducing inducer concentrations or switching to weak inducers, e.g. lactose for the T7 promoter [132,139,140].

If transcription efficiency limits the final yield, use of well-expressing genes in bicistronic expression constructs might be exploited to increase mRNA levels [141]. Efficiency of transcription initiation can be mediated by adapting the codon usage of the 5' region of the recombinant gene to the host's own one, but can also be improved by fusing well-expressed genes, encoding for e.g. β-galactosidase upstream of the gene of interest [142].

Another way to guide the protein along the secretory route to the plasma membrane is the use of N-terminal, host-specific secretory targeting sequences, e.g. of the Ste2 receptor [88,143], the prepropeptide of the *S. cerevisiae *α-mating factor or the signal peptide of acid phosphatase [26,135-138,144-149]. The majority of integral membrane proteins (IMPs) however possesses a native N-terminal signal sequence, thus the use of N-terminal tags - either for detection by immunoblotting or for purification - might lead to delusive conclusions due to processing of the signal peptide [79]. When information on protein topology is scarce, the use of N-terminal secretory signal sequences might also interfere with protein folding and insertion into the membrane. This strategy, however, usually worked for GPCRs, where the N-terminus is facing the outward side of the membrane [150]. Apart from signal sequences, also N- or C-terminal fusions with soluble cytoplasmic (GFP, glutathione S-transferase) and periplasmic proteins (maltose binding protein) may improve expression of otherwise poorly expressed targets and promote their stabilization in *E. coli*, putatively by protecting membrane proteins against proteolytic degradation of vulnerable N- or C-termini [151-155]. In this context, targeting to and integration into bacterial membranes has also been facilitated by fusion to other membrane proteins (maltoporin, glycerol-conducting channel GlpF, mistic) [142,156]. Due to its ability to integrate into the membrane independently of the translocon, mistic, an integral 13 kDa membrane protein derived from *Bacillus subtilis*, can be used to eliminate bottlenecks encountered therein [157,158]. Evidently, topologies of both membrane proteins - target protein and fusion partner - have to be considered, e.g. the N-terminal fusion of GlpF is limited to membrane proteins with N-termini facing the cytoplasm [156].

Finally, external parameters considerably affect transcription and translation rates, cultivation temperature being recognized as the most effective one. Generally, best growth conditions in terms of biomass yield are not necessarily ideal for heterologous protein expression, but might rather elicit a stress response [159] or result in low yield of functional membrane protein [144,160]. In fact, folding and membrane insertion of the protein can be promoted by lowering the cultivation temperature [140,144,149,160,161]. At low temperature the activity of the transcriptional and translational apparatus is reduced, avoiding an overload thereof. Additionally, many cold-shock chaperones are induced at lower temperature, which promotes folding [162], but also reduces proteolytic degradation [163,164]. The positive effect might further be linked to higher protein stability at low temperatures, but also to decreased folding stress. Especially in prokaryotes, folding of eukaryotic membrane proteins benefits from reduced transcription and translation rates as a result of adaptation to eukaryotic translation rates.

Effects of other external parameters, i.e. pH, osmolarity and aeration, on membrane protein expression have not been studied thoroughly so far. Only the influence of pH on the yield of individual membrane proteins has been documented for yeast, suggesting optimal values ranging from neutral to alkaline pH [160,161]. These studies can not serve as general guidelines, however. Optimization is required for each target protein.

##### 2.2.2. Relieving folding and translocation stress

**Figure 1:**
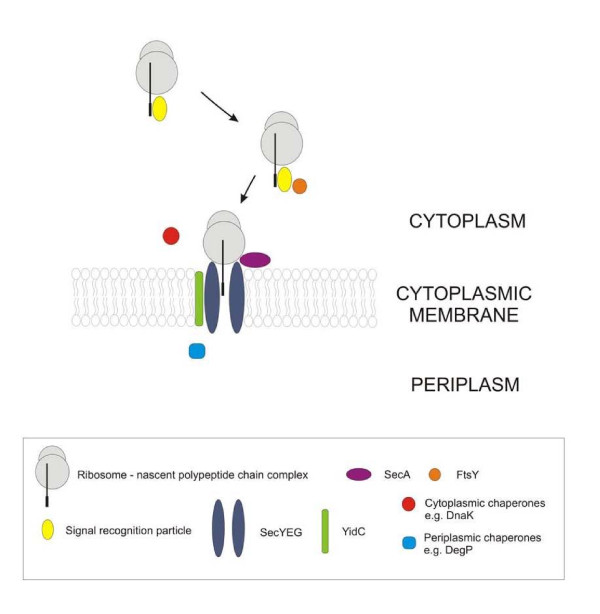
**Membrane protein biogenesis in prokaryotes**. In prokaryotes, most membrane proteins are targeted to and inserted into the cytoplasmic membrane by the SRP pathway, which involves interaction of the growing polypeptide chain with the signal recognition particle (SRP) and its receptor FtsY, binding of the nascent polypeptide chain-ribosome-complex to the SecYEG/YidC pore, translocation of cytoplasmic and periplasmic loops across the cytoplasmic membrane and their folding by SecA and various chaperones and insertion of hydrophobic segments into the membrane. The autonomous, YidC and Tat pathways, that are used by small proteins and membrane-associated periplasmic proteins, respectively, are mentioned here for sake of completeness.

**Figure 2:**
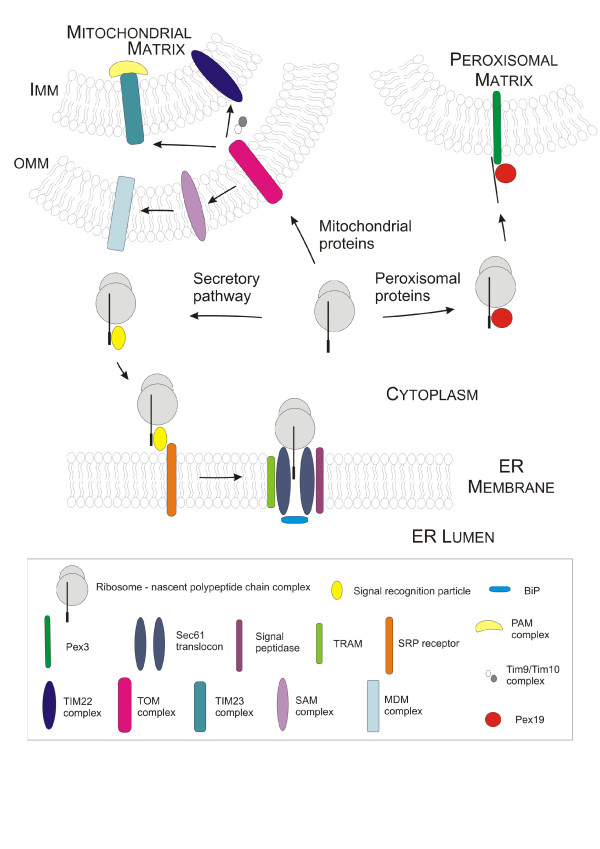
**Membrane protein biogenesis in eukaryotes**. In eukaryotic cells, membrane protein biogenesis occurs in a cotranslational way. Proteins residing in membranes of ER and Golgi apparatus or in plasma membrane use the secretory pathway. Like in prokaryotes, SRP recognizes polypeptides protruding from the ribosome complex, thereby transiently attenuating translation. As soon as the SRP-ribosome complex interacts with the SRP receptor and docks to the Sec61 translocon pore, translation resumes, BiP relocates, thereby opening the lumenal gate and the membrane protein enters the membrane by lateral diffusion through the Sec61 pore. Peroxisomal membrane proteins either use the Pex19/Pex3-mediated way (class I proteins, shown here) or are thought to reach the peroxisomal membrane via the ER (class II) [208]. Mitochondrial membrane proteins pass through the outer mitochondrial membrane (OMM) via the TOM complex (translocase of the outer membrane). While β-barrel proteins of the OMM are transported to the SAM and MDM complexes (sorting and assembly machinery), the TIM22 and TIM23 complexes (translocase of the inner mitochondrial membrane) are used to target proteins to the inner mitochondrial membrane [209].

The gross majority of membrane proteins follow the same route like soluble, secretory proteins once the nascent polypeptide emerges from the ribosome, including targeting to the translocon via the SRP particle (Figure 1, Figure 2). In contrast to secretory proteins, however, the most prominent bottleneck in overexpression of membrane proteins is the physical space within or at a lipid bilayer. Difficulties arise from limitations in membrane capacity when accommodating additional proteins. Each membrane has an optimal ratio between lipids and membrane proteins. Thus, variations caused by massive protein insertion affect membrane integrity and cell functionality [165]. Upon disturbance of this balance, expression systems often react with stress responses including protein degradation.

Prokaroytes

In prokaryotes, the SRP guides a nascent polypeptide chain to its membrane-associated receptor FtsY and subsequently to the Sec translocon consisting of the protein conducting channel SecY, SecE and SecG [166]. Sec translocon-mediated insertion into the cytoplasmic membrane is supported by the peripherally associated ATPase SecA, which is required for translocation of periplasmic loops and secretory proteins, and by YidC, which is thought to mediate lateral transport of transmembrane helices (TMH) into the membrane (Figure 1). Two proteases residing in the cytoplasmic membrane, FtsH and HtpX, play a crucial role in quality control of membrane proteins [167].

During membrane protein production, enhanced levels of folding proteins might be required in order to cope with the membrane protein load. As a shortage of foldases might limit the cellular productivity, membrane protein production can be optimized by providing surplus compounds of the assembly machinery. The effect of coexpression and deletion of chaperones and proteases has been recently investigated for *E. coli *expressing GPCRs [168,169]. Using the human cannabinoid receptor CB1 as model protein, a transposon library of *E. coli *was screened for increased expression levels [169]. Inactivation of DnaJ (*dnaJ*::Tn5) resulted in a higher abundance of membrane-integrated receptor and also abolished growth-retarding effects of overexpression. On the other hand, coexpression of DnaJ/K during expression of the magnesium transporter CorA increased expression levels, and its role in membrane protein assembly has been ascribed to its capability to prevent accumulation of the protein in inclusion bodies [170]. From X-ray structural analysis, evidence emerged that presence of a large extramembraneous domain requires an increased amount of chaperones to mediate its folding [171]. The exact role of DnaJ during CB1 expression is not clear. According to topology predictions, the receptor lacks any large loops connecting its 7 TMHs. However, the N-terminal 116 amino acids were found to be located on the extracellular side, while the shorter C-terminal domain faces the cytoplasm. It has thus been speculated that the inhibitory role of DnaJ is linked to its ability to interact with the N-terminus proceeding from the ribosome, thus preventing its insertion into the Sec translocon [169]. Given that only total amounts of receptor are indicated, it remains to be clarified, whether non-functional and, thus, non-toxic protein has accumulated in the membrane due to absence of folding chaperones or whether the positive effect of dnaJ knockout is due to its strong interaction with the N-terminal region. Apart from dnaJ, positive effects on membrane protein biogenesis have also been observed upon inactivation of the transcription factor nhaR, which regulates expression of genes involved in cation transport and of the DNA damage-inducible helicase dinG. Yet, their precise role during membrane protein biogenesis need to be elucidated in detail [169]. Judging from the expression yields obtained for other membrane proteins (human CB2 receptor, human bradykinin receptor 2, human neurokinin receptor 1), the length of the N-terminal tail seems to play a crucial role, as no improvement has been achieved by transposon-mediated dnaJ inactivation.

Another study addressed the effect of coexpression of different chaperones, translocon compounds and proteases on membrane protein biogenesis, exemplified for the central and peripheral cannabinoid receptor (CB1 and CB2), the bradykinin receptor 2 (BR2) and the neurokinin-(substance 1) receptor 1 (NKR1) [168]. While many chaperones including the membrane integrase YidC, SRP compound 4.5S RNA, SRP receptor FtsY, compounds of the translocon SecY and SecE and the chaperones SecB, GroEL/GroES did not promote membrane protein production, a moderate increase in expression levels of CB1 has been achieved by coexpression of the SRP compound Ffh, chaperones DnaJ/K and the peptidyl-prolyl isomerase trigger factor Tig - thus opposing the previous study [169]. Unexpectedly, highest expression levels were obtained upon coexpression of FtsH, a membrane-anchored AAA+ protease [169]. Besides improved amounts of membrane-inserted receptor, a two-fold higher cell density provided evidence that cells experienced less stress, although cell division was impaired as shown by cell elongation. The fact that no improvement in binding efficiency of GPCRs was achieved and strains reached higher cell densities compared to control strains left a few questions unanswered. FtsH is involved in quality control of membrane proteins, however no effect has been observed for other AAA+ proteases (HtpX, YcaL, YfgC, and YggG). Preliminary results of a transcriptome analysis indicated that glycerol metabolism was largely rearranged in mutant strains, suggesting that the lipid composition of cytoplasmic membranes was altered [168]. Recent studies examined the stress response upon CB1 or FtsH production and revealed an induction of genes from the σ^32 ^heat shock regulon [172]. Using promoter-GFP fusions, it was demonstrated that coexpression of CB1 and FtsH resulted in additive stress responses as assessed by flow cytometry, clearly indicating that FtsH is not able to attenuate stress responses caused by CB1 expression. Instead, stress-related effects were boosted further by FtsH coexpression. Authors proposed that this protease likely proactively prepares the cells to cope with the toxic effects caused by CB1.

Eukaryotes

Typically, membrane proteins - excluding proteins destined for peroxisomal, chloroplast and mitochondrial membranes - enter the secretory pathway by translocating into the ER membrane where folding and maturation of the proteins take place [68,173,174]. Like in prokaryotes, as soon as the signal sequence protrudes from the the growing polypeptide-ribosome complex, the SRP guides the complex to the ER membrane (Figure 2). Translation resumes when the complex docks to the Sec61p pore, triggering opening of the lumenal BiP-mediated gate. Yeast cells deploy several mechanisms to cope with the increased membrane protein load. Overproduction of ER-resident membrane proteins triggers enhanced proliferation of ER membranes as shown for HMG-CoA reductase and CYP52 A3, mediated by induction of the unfolded protein response [175-177]. At the same time, ER chaperones act in concert to properly assemble membrane proteins or promote folding of lumenal, extramembraneous domains, thus their coexpression can boost ER folding capacity. Previously, expression levels of a serotonin transporter in the baculovirus expression system were improved by a factor of three upon coexpression of the ER-resident chaperone calnexin [178]. As this membrane protein requires the presence of a distinct N-glycan moiety to mature into functional protein, increased levels of calnexin were required to ensure efficient N-glycosylation within the ER. Coexpression of calreticulin and BiP also increased the final yield, albeit to a lesser extent. Similarly, increased abundance of the dnaK-type chaperone BiP mRNA has been found during overexpression of cytochrome P450 52A3 in *S. cerevisiae *[176,177] and of the 2-compound cytochrome P450 system including P450 reductase and benzoate p-hydroxylase BphA in *A. niger *[179]. On the other hand, deletion of Cne1, a yeast homolog of mammalian calnexin and calreticulin, resulted in higher levels of human transferrin receptor in *S. cerevisiae *[180]. As part of the ER quality system, calnexin was thought to prevent transport of the human transferrin receptor to the plasma membrane, which was then reversed by CNE1 deletion. Furthermore, increased levels of Sec61 translocon have been observed during membrane protein production [177]. Folding of transmembrane segments is largely mediated by membrane-resident chaperones, which often display a high substrate specificity. Folding of amino acid permeases, hexose transporters, phosphate transporters and chitin synthase III was facilitated by chaperones Shr3, Gsf2, Pho86 and Chs7 respectively [181-183]. Deletion of the respective chaperone resulted in protein aggregation and failure of proteins to exit the ER [182].

Besides mediating membrane protein folding, the ER serves as a quality checkpoint to control protein integrity before the protein exits the ER. If cellular capacities to correctly assemble membrane proteins are exhausted, aberrant protein accumulates within the ER and is subjected to different rescue or degradation routes. Eukaryotic cells employ different strategies to cope with the accumulation of misfolded proteins within the ER. First, inhibition of translation initiation counteracts further accumulation of unfolded polypeptides. Secondly, under conditions of increased membrane protein load, cells overcome folding limitations by upregulating expression of chaperones, thereby increasing the folding capacity within the ER [184]. The increased chaperone requirement in turn often triggers induction of the UPR, an intracellular signalling pathway that acts to relieve stress situations [185,186]. Finally, proteins that fail to reach their native fold are targeted to the proteasome for degradation, through dislocation to the cytoplasm and protein ubiquitination. Depending on the topology and location of the misfolded domain, different pathways are employed for membrane protein turnover. While proteins with large cytosolic domains enter the Doa10-dependent route, lumenally exposed lesions target the protein to the Hrd1 degradation pathway [187-191].

Being an indicator for folding problems and ER stress, the UPR has also been rationally exploited as a sensing mechanism allowing optimized production of functional membrane proteins in *S. cerevisiae *[186]. The trypanosomal H^+^/adenosine cotransporter was used as model protein and β-galactosidase as reporter with a transcriptional UPR element driving expression of the lacZ gene. Expression conditions were optimized for the transporter by minimizing UPR activation. This strategy, however, only works for membrane proteins that elicit the UPR upon overproduction as observed for evolutionarily divergent proteins, while most proteins from more closely related organisms, e.g. yeast receptor and plant transport proteins, did not highlight any folding problems.

##### 2.2.3. Increasing protein stability by chemical chaperones

Membrane proteins often associate with specific compounds *in vivo*, be it defined lipids or interacting proteins, and their presence is crucial for proper folding, activity and/or stability. In this context, the subcellular localization plays a significant role. Many mitochondrial membrane proteins tightly interact with cardiolipin, as shown by their association in protein crystals even after the protein has been subjected to several purification steps [192,193]. Thus, shortage or even lack of defined lipid molecules may affect heterologous expression of membrane proteins. Lipid compositions vary between different hosts and organelles (reviewed by [36]). For full activity, it is often crucial to keep the membrane protein in a similar environment as in its native host, e.g. by targeting it to the proper organelle or by choosing a related host. Membrane proteins may assemble into supercomplexes *in vivo *[194]. Thus, co-expression of proteins that are known to form stable complexes or tightly interact with the target protein might also improve overexpression. For example, the coexpression of human β_2_-adrenergic receptor and its corresponding mammalian G protein subunit in *S. cerevisiae *did not only improve stability, but was also necessary for *in vivo *functionality [143]. Similarly, small chemical ligands can reduce conformational flexibility by tight association, and thus decrease the likelihood of hydrophobic domains to be exposed and attacked by proteases or becoming prone to aggregation. This idea has been successfully applied in heterologous expression of GPCRs. By addition of specific ligands at concentrations close to 100 times the K_d _value during the induction phase, expression levels and stability of GPCRs were increased [135,144,149].

Apart from specific ligands, chemical supplements, e.g. DMSO, glycerol and histidine, are known to affect membrane protein expression positively as evaluated extensively for GPCRs [135,144,195]. The stimulating effect of DMSO at low concentrations (2.5% (v/v)) seems to be linked to its ability to modify the physical properties of membranes and membrane capacity by upregulating transcription of genes involved in lipid biosynthesis [196]. DMSO also increases the permeability of membranes and, thus, improves the access of externally added ligands to membrane proteins [197]. Like for many chaperones, use of DMSO is not a universal key to success, as adverse effects including downregulated expression and proteolysis have also been observed for plasma membrane transporters in yeasts [198]. Chaperoning activities and stabilization of protein conformation, especially of glycoproteins, have been ascribed to glycerol. Addition of 10% (v/v) glycerol during growth led to an increase in expression levels, exemplified for a human P-glycoprotein during heterologous expression in *S. cerevisiae *[195]. Glycerol was thought to directly increase stability of the membrane protein. The positive influence of histidine, which is most often added at concentrations of 0.04 mg mL^-1^, is thought to be related to its ability to act as a physiological antioxidant, but the trivial possibility of improved availability of this amino acid can not be ruled out [199].

##### 2.2.4. Rationalizing membrane protein production by engineering host cells

As membrane protein biogenesis requires a tight balance between different processes and even small changes might have far-reaching consequences the application of generally valid engineering approaches is complicated. In the past, attention had been chiefly drawn to modifications of cultivation conditions or of expression constructs, i.e. gene dosage, promoters, purification tags, leader sequences, fusion proteins, in order to optimize membrane protein production. If expression failed in an expression system a different expression host was investigated for its ability to correctly synthesize the protein. Experience has shown that many years of effort are necessary in order to succeed in protein crystallization. Many improvements have been achieved on a trial-and-error basis. Unfortunately, they did not provide any general guideline for membrane protein production, not to mention any insights into the physiological constraints host cells encounter. There are no great endeavours to adapt microbial expression hosts to membrane protein production. Instead, many labs have resorted to well-established protease-deficient strains in order to minimize proteolytic degradation rather than alleviating host-specific bottlenecks [77,79,144].

Low success rates and protein aggregation have sparked interest in the mechanisms and physiological effects of membrane protein biogenesis allowing identification of putative bottlenecks. Analysing the consequences of membrane protein production from a comprehensive molecular and cell biological view - accomplished on the transcriptome and/or proteome level - allows to study physiological effects, cellular constraints and stress regulation. Most importantly, the -omics approach will help to apply the compiled knowledge for rational engineering of the host strain. Although no general, single-key bottlenecks in membrane protein expression are known so far, factors like chaperones, foldases, proteases etc., which were found to facilitate membrane protein expression in single cases, are in focus of comprehensive molecular analyses of membrane protein overexpressing hosts by transcriptomics or proteomics. Several groups have already addressed the question whether genetic engineering of the host, going beyond simply shifting the rate-limiting step within one single metabolic pathway or process, can relieve specific bottlenecks.

2.2.4.1. Classical engineering

The traditional way to improve heterologous hosts in terms of productivity is the classical strain engineering, involving chemical mutagens or UV radiation. In fact, so far only *E. coli *BL21(DE3) has been tailored for improved membrane protein production capacity by classical selection more than one decade ago [39]. After having recognized that overexpression of an oxoglutarate-malate carrier protein negatively affected cell growth, strains that were able to survive during protein expression were selected, termed Walker strains or C41(DE3) and C43(DE3). Interestingly, in these strains higher expression levels and unimpaired growth were observed upon overexpression of soluble and membrane proteins that otherwise hampered cell viability of BL21(DE3). Miroux and Walker also noted that lower transcript levels had been reached by the mutant strains compared to the BL21(DE3), suggesting that transcriptional rates had slowed down. Later, it was postulated that the high success rate of Walker strains was linked to their ability to proliferate additional intracellular membranes and, thus, tolerate increased membrane protein loads [54]. In order to decipher the mechanisms, which make these strains superior to conventional ones for membrane protein production, the proteome of the Walker strains overexpressing the prokaryotic membrane protein YidC was compared to the one obtained from the parental strain BL21(DE3) [38].

Although the Walker strains behaved superior regarding growth characteristics and final expression levels, the same proteome response was observed in all strains, thus giving no clue what was the key to success in the Walker strains. However, lower abundance of chaperones and proteases (ClpB, IbpA, HslUV) involved in resolving cytosolic aggregates indicated that in contrast to BL21(DE3) the Walker strains experienced fewer problems in folding and insertion of membrane proteins at the Sec translocon. Driven by many open questions, the de Gier group analyzed the genetic features of the strains in detail and discovered that the high success rate of the Walker strains in membrane protein expression likely originates from a lower transcriptional rate caused by reduced abundance of T7 RNA polymerase, which is used in these strains to drive recombinant protein expression. Mutations in the lacUV5 promoter, a strong variant of the wild-type *lac *promoter driving expression of the T7 RNA polymerase, have accumulated in these strains which decrease the promoter strength. As a result, transcription, translation and protein insertion into the membrane are well balanced. Cells more easily cope with the membrane protein load and accomodate them in a functional way into the cytoplasmic membrane [38]. The mutations leading to lower expression rate accumulated in the -10 region thereby reconverting the lacUV5 variant to the wildtype *lac *promoter, and in the lac operator region making the promoter susceptible to catabolite repression by glucose. The latter resulted in delayed expression. In turn, the de Gier lab has constructed engineered strains, designated Lemo21(DE3), which allow to control activity of T7 RNA polymerase by its inhibitor T7 lysozyme (T7Lys). In these strains, coexpression of T7Lys under control of the titrable rhamnose inducible promoter rhaBAD is exploited to rationally dampen T7 RNA polymerase activity. This strategy allows to fine-tune expression of soluble and membrane-embedded protein of both prokaryotic and eukaryotic origin in *E. coli *by avoiding conditions which result in aggregation of protein and recruitment of chaperones and proteases. Once again this study demonstrated that slowing down transcription is one key to success for membrane protein biogenesis.

2.2.4.2. The use of -omics technologies to rationalize membrane protein production

Prokaryotic systems

Motivated by poor knowledge on the host's physiological response to membrane protein production, Wagner and coworkers systematically analysed the effect of overexpression of three different membrane proteins (YidC, YedZ, LepI) on the proteome of *E. coli *[200]. Overexpression of all model proteins hampered growth, resulting in early transition to stationary phase. According to flow cytometry, cells were slightly bigger, indicating increased proliferation of additional membranes to host foreign membrane proteins, but also impaired cell division. Other studies have also pointed out that *E. coli *cells overcome physical limitations by proliferating additional membranes in order to cope with the increased membrane protein load [201]. Judging from the proteome of cell lysate, protein aggregates and cytoplasmic membranes, it became clear that massive expression of membrane proteins severely disturbed protein homeostasis in the cytoplasm of *E. coli *[200]. In production strains, overloading of the translocon entailed an accumulation of cytoplasmic aggregates, thereby evoking sequestration of chaperones like DnaJ/K and GroEL/S as shown by immunoblotting. Increased abundance of proteolytic proteins (HslU/V, ClpXP, ClpB; assisting proteins IbpA/B) combined with higher levels of chaperones suggested that in response to membrane protein misfolding the heat shock reponse is activated. The recruitment of chaperones and proteases was required to reduce inclusion body formation and resolve overexpressed protein that had accumulated in inclusion bodies. As revealed by transcriptome analysis, transcription of heat shock genes (lon, ClpP, HslU/V) and molecular chaperones (DnaK, DnaJ) was upregulated in response to inclusion body formation [202,203].

Besides the overexpressed membrane proteins, cytoplasmic aggregates contained chaperones, proteases (HslU/V, ClpXP), many essential cytoplasmic proteins and many precursors of periplasmic and outer membrane proteins, indicating that the capacity of the translocation machinery was saturated [200]. Therefore, coupling of transcription, translation and targeting needs to be properly balanced. In this specific case, protein aggregation was probably also evoked by the use of the strong promoter of the T7 polymerase which was employed to drive expression of the membrane proteins. The idea that membrane protein production exceeded the capacity of the Sec translocon was confirmed by different observations. On the one hand, secretion efficiency was reduced suggesting that cotranslational membrane protein biosynthesis competed with the posttranslational process of protein secretion. On the other hand, low levels of endogenous membrane proteins provided evidence that recombinant membrane protein outcompeted endogenous protein production, i.e. most prominently respiratory chain complexes. Increased levels of translocon components SecY, SecE and SecG and membrane-associated ribosomal subunit L5 however, inferred a saturation of the translocation capacity. Membrane protein overproduction compromised cellular respiration, as shown by reduced oxygen uptake rates and low cytochrome c oxidase activity. As a consequence of inefficient energy metabolism, the Arc two-component system was activated and the acetate-phosphotransacetylase pathway was primarily employed for ATP production. Interestingly, expression of all model proteins evoked similar responses on the proteome level, suggesting that *E. coli *faces similar bottlenecks during membrane protein expression irrespective of the protein. This sounds feasible as membrane proteins are typically overexpressed in the plasma membrane in prokaryotes, while in eukaryotic cells membrane proteins pass through and end up in different organelles and, thus, optimization approaches depend on the subcellular localization. It should be emphasized that depending on protein structure different, translocon-independent pathways might be used for targeting and membrane insertion in prokaryotes as observed for the magnesium transporter CorA [171]. This work also carries great promise for future host engineering and holds out the prospect that obstacles encountered in membrane protein production can be alleviated on a broad basis and do not require optimization for each individual protein. Based on the existing data, compounds of the translocon machinery are promising candidates for cell engineering with the aim to obtain higher productivity. Although not yet demonstrated, the authors also suggested the FtsH protease as putative target for knockout or inhibition. Alternatively, coexpressing FtsH inhibitors, e.g the peptide SpoVM derived from *Bacillus subtilis*, might improve membrane protein overexpression. However, this would conceptually contradict the unexpectedly positive effects of FtsH overexpression [169].

Yeast cells

The physiological response of yeast to membrane protein production has hardly been deciphered in a rational way. Some early studies aimed at considering membrane protein production in relation to cell physiology with the aim to minimize folding stress within the ER by circumventing induction of the UPR [186]. In order to reveal which factors govern membrane protein degradation and whether ER-localized membrane proteins follow the ERAD degradation route, a recent study analyzed the transcriptome of *S. cerevisiae *cells overexpressing a non-functional variant of the 12 TMH ABC transporter Ste6 [204]. Due to improper folding of the extramembraneous cytoplasmic domain and the transmembrane segments the protein accumulated within the ER membrane and was subjected to proteolytic degradation. The transcript profile provided evidence that the transcription factor Rpn4 played a prominent role in combating stress evoked by accumulation of misfolded membrane protein in the ER. Authors proposed a similar role for Rpn4 in degradation of ER-resident membrane proteins as attributed to UPR-transcription factor Hac1. During stress situations, the transcription factor Rpn4 regulates the expression of proteasomal genes and, thus, ensures survival of the cell.

Other studies have emphasized that conditions that are optimal for growth and high transcriptional rates rarely coincide with those for production of functional membrane proteins, which motivated Bonander and coworkers to study the differences between cultivation conditions in detail [160]. As target protein, the glycerol uptake/efflux facilitator protein Fps1p was homologously overexpressed in *S. cerevisiae*, and effects of temperature and pH on expression levels and mRNA availability were investigated. The study demonstrated that conditions favouring fast growth rate and high transcriptional activity do not promote membrane insertion of aquaglyceroporin Fps1 [160]. Improved levels of membrane-bound protein were achieved by shifting temperature to 20°C, thus slowing down growth rate. This was another piece of evidence that gene dosage and transcript abundance do not limit membrane protein expression. Motivated to decipher which mechanisms govern membrane protein biogenesis at different temperatures and pH, transcriptomes of expression strains cultivated at 30°C (pH 5) and 35°C (pH 5 and pH 7) were compared. Already a temperature shift of 5°C had a strong impact on the transcriptome, especially on transcript abundance of proteins involved in secretion and yeast cellular physiology. Recently, also data for the most productive 20°C-grown cells were published [224]. Downregulation of genes encoding for intracellular protein trafficking (*SEC62, APM3 *- mediating Golgi-to-vacuole transport and vacuolar trafficking, *VTC3 *- vacuolar trafficking) and proteins involved in ribosome biogenesis and assembly (*RPP1A, CGR1, BMS1*) indicated that the secretory pathway was compromised [205]. From higher abundance of *SRP102 *transcripts encoding β subunit of SRP authors concluded that the translocon was overloaded. In response to heterologous membrane protein synthesis, sterol biosynthesis was found to be upregulated as indicated by increased levels of *ERG9*, while transcription of the heat shock protein *HSP82 *was induced in response to growth at 35°C. Increased levels of *HSP82*, however, might have been also required for correct insertion of membrane proteins. Following initial transcriptome analysis, authors investigated by which means expression of Fps1p can be improved in *S. cerevisiae *[206]. Among differently regulated and related genes, 43 genes were selected as initial targets to assess membrane protein biogenesis in respective deletion or overexpression strains. Assuming that the Sec translocon capacity was limited effects of *SEC63 *coexpression and *SRP102 *deletion were investigated, but no improvements were obtained [200]. Strongest effects were observed when the Fps1p channel was produced in yeast lacking compounds of the transcriptional Spt-Ada-Gcn5-Acetyl (SAGA) transferase (SPT3, GCN5) and co-activator complex (SRB5), yielding up to 70-fold improved Fps1p levels. The 1.8 MDa-complex is involved in regulation of RNA polymerase II-mediated transcription and provides an alternative route in recruiting the transcription machinery to promoters independently of general transcription factors [207]. The high membrane protein yield was found to be closely linked to an increase in *BMS1 *levels compared to the wildtype strain. Being a nucleolar protein, *BMS1 *plays a role in ribosome biogenesis regulating biosynthesis of the 40S subunit. In turn, authors investigated effects of Bms1p abundance on Fps1p production by employing the doxycycline-dependent titratable Tet promoter to adjust *BMS1*. An improvement of up to 137-fold was achieved when 5 times more *BMS1 *was provided compared to the wildtype. Similar effects were observed for other membrane proteins (human A_2a _receptor) and soluble proteins (GFP).

Yields of functional membrane and soluble proteins were also increased by altering the ratio of 60S and 40S ribosomal subunits from 1:1 to 2:1 via *BMS1 *regulation, corresponding to a shift in 25S to 18S ratio from 2:1 to 3:1. As shown by polysome profiling, higher levels of free 60S subunit were present in overexpressers, while 40S and 80S levels remained constant emphasizing that Bms1p regulates biosynthesis of the 60S subunit. The improved strains exhibited a slower growth rate, but were metabolically more efficient compared to standard strains as revealed by microcalorimetry. Ribosome biogenesis is one of the major consumers of cellular energy. By tuning BMS1 transcript levels in a doxycyclin-dependent manner yields of both membrane and soluble proteins were optimized, indicating, that each protein requires individual adjustment of the doxycyclin concentration and thus BMS1 levels to achieve best expression levels [206].

## Conclusions

The past has shown that membrane proteins are not only tricky to handle once they have been solubilized, but first of all they are difficult to obtain in large amounts - even by overexpression in heterologous hosts. Fortunately, published work provides insights into the mechanisms of membrane protein biogenesis so that many useful guidelines, hints and tips are available enabling us to improve the design of future experiments. Expression systems that are closely related to the target protein from a phylogenetic point of view provide a similar cellular milieu as the protein's native host. Accordingly, this holds also true for lipid compositions of membranes and protein processing machineries. Many recent advances in understanding membrane protein biogenesis and the role of specific lipids in correct folding and topology suggest that homologous overexpression is most promising to finally succeed in structure determination. An estimated 45% of all structures originate from homologous overexpression, thus homologous or closely related hosts have proven most feasible for membrane protein production [117]. Microbial systems, especially prokaryotic ones, in many cases might not fulfil the requirements for eukaryotic membrane proteins. However, membrane protein expression can be tuned by other means including gene design or slowing down transcriptional and translational rates by promoter and host engineering to match the ideal folding and assembly rates. One key strategy to remedy misfolding within the cell takes advantage of increased amounts of chaperones, thus guiding membrane proteins along its biogenesis and folding process.

The era of genomics has brought many exciting advances in membrane protein research. The available -omics technologies provide a complementary approach to decipher mechanisms underlying membrane protein biogenesis. By exploring the host-related molecular and physiological response, target proteins can be selected for rational host engineering. An exciting example is a recent report on the expression of eukaryotic membrane proteins in *S. cerevisiae*, allowing to obtain large amounts of both membrane-embedded and soluble proteins by tuning composition of ribosomal subunits [206]. Our basic understanding of membrane protein biogenesis is continuing to expand and thus offers exciting opportunities for future work. The breadth of recent progress in elucidation of membrane protein structure determination provides evidence that exciting years in membrane protein research lie ahead.

## List of Abbreviations used

CYP: cytochrome P450; DMSO: dimethyl sulfoxide; ER: endoplasmic reticulum; FACS: Fluorescence Activated Cell Sorting; GAL1: galactose kinase; GAL10: UDP-glucose 4-epimerase; GFP: green fluorescent protein; GPCR: G protein coupled receptor; HTP: high throughput; IMP: integral membrane protein; NMR: nuclear magnetic resonance; PTM: posttranslational modification; SRP: signal recognition particle; TMH: transmembrane helix; UPR: unfolded protein response.

## Competing interests

The authors declare that they have no competing interests.

## Authors' contributions

AG and MF suggested and defined the topic of this review article. MF drafted the manuscript, HP and AG revised it critically. All authors read and approved the final manuscript.
